# Growth of *Enterococcus faecalis* ∆*plsX* strains is restored by increased saturated fatty acid synthesis

**DOI:** 10.1128/msphere.00120-23

**Published:** 2023-06-08

**Authors:** Qi Zou, Huijuan Dong, John E. Cronan

**Affiliations:** 1 Department of Biochemistry, University of Illinois at Urbana-Champaign, Urbana, Illinois, USA; 2 Department of Microbiology, University of Illinois at Urbana-Champaign, Urbana, Illinois, USA; The University of Iowa, Iowa City, Iowa, USA

**Keywords:** phospholipid synthesis, growth, suppressor, saturated fatty acids, *sn*1-position

## Abstract

The *Enterococcus faecalis* acyl-acyl carrier protein (ACP) phosphate acyltransferase PlsX plays an important role in phospholipid synthesis and exogenous fatty acid incorporation. Loss of *plsX* almost completely blocks growth by decreasing *de novo* phospholipid synthesis, which leads to abnormally long-chain acyl chains in the cell membrane phospholipids. The ∆*plsX* strain failed to grow without supplementation with an appropriate exogenous fatty acid. Introduction of a ∆*fabT* mutation into the ∆*plsX* strain to increase fatty acid synthesis allowed very weak growth. The ∆*plsX* strain accumulated suppressor mutants. One of these encoded a truncated β-ketoacyl-ACP synthase II (FabO) which restored normal growth and restored *de novo* phospholipid acyl chain synthesis by increasing saturated acyl-ACP synthesis. Saturated acyl-ACPs are cleaved by a thioesterase to provide free fatty acids for conversion to acyl-phosphates by the FakAB system. The acyl-phosphates are incorporated into position *sn*1 of the phospholipids by PlsY. We report the *tesE* gene encodes a thioesterase that can provide free fatty acids. However, we were unable to delete the chromosomal *tesE* gene to confirm that it is the responsible enzyme. TesE readily cleaves unsaturated acyl-ACPs, whereas saturated acyl-ACPs are cleaved much more slowly. Overexpression of an *E. faecalis* enoyl-ACP reductase either FabK or FabI which results in high levels of saturated fatty acid synthesis also restored the growth of the ∆*plsX* strain. The ∆*plsX* strain grew faster in the presence of palmitic acid than in the presence of oleic acid with improvement in phospholipid acyl chain synthesis. Positional analysis of the acyl chain distribution in the phospholipids showed that saturated acyl chains dominate the *sn*1-position indicating a preference for saturated fatty acids at this position. High-level production of saturated acyl-ACPs is required to offset the marked preference of the TesE thioesterase for unsaturated acyl-ACPs and allow the initiation of phospholipid synthesis.

## INTRODUCTION

Phospholipids are essential components in cell membrane formation and function (1 - 3). Phospholipid synthesis requires successive acylation of the *sn*1- and *sn*2-positions of *sn*-glycerol-3-phosphate (G3P) to generate phosphatidic acid followed by a series of reactions that produce various phospholipid species (2). In *Escherichia coli* and other γ-proteobacteria, the PlsB G3P acyltransferase catalyzes acylation at the *sn*1-position of G3P using acyl-acyl carrier protein (ACP) or acyl-coenzyme A (CoA) as the acyl donor. The 1-acyl-G3P acyltransferase, PlsC, then transfers fatty acyl chains to the *sn*2-position to produce phosphatidic acid (2). However, in Firmicute bacteria such as *Bacillus subtilis*, *Staphylococcus aureus*, *Streptococcus pneumoniae,* and *Enterococcus faecalis*, acylation of the *sn*1-position of G3P is catalyzed by the G3P acyltransferase PlsY, which requires acyl-phosphates (acyl-PO_4_) as the acyl donor (1). The *de novo* synthesis of this intermediate relies on the acyl-ACP-phosphate acyltransferase, PlsX, which converts acyl-ACP to acyl-PO_4_ and thereby coordinates fatty acid synthesis with phospholipid synthesis (1, 2). In the presence of exogenous fatty acids, which are converted to acyl-PO_4_ species by the fatty acid kinase (FakA/B) pathway (4), PlsX converts acyl-PO_4_ to acyl-ACP to allow PlsC catalyzed acylation of the *sn*2-position of 1-acyl-G3P to give the phosphatidic acid required for phospholipid synthesis (2).

**Fig 1:**
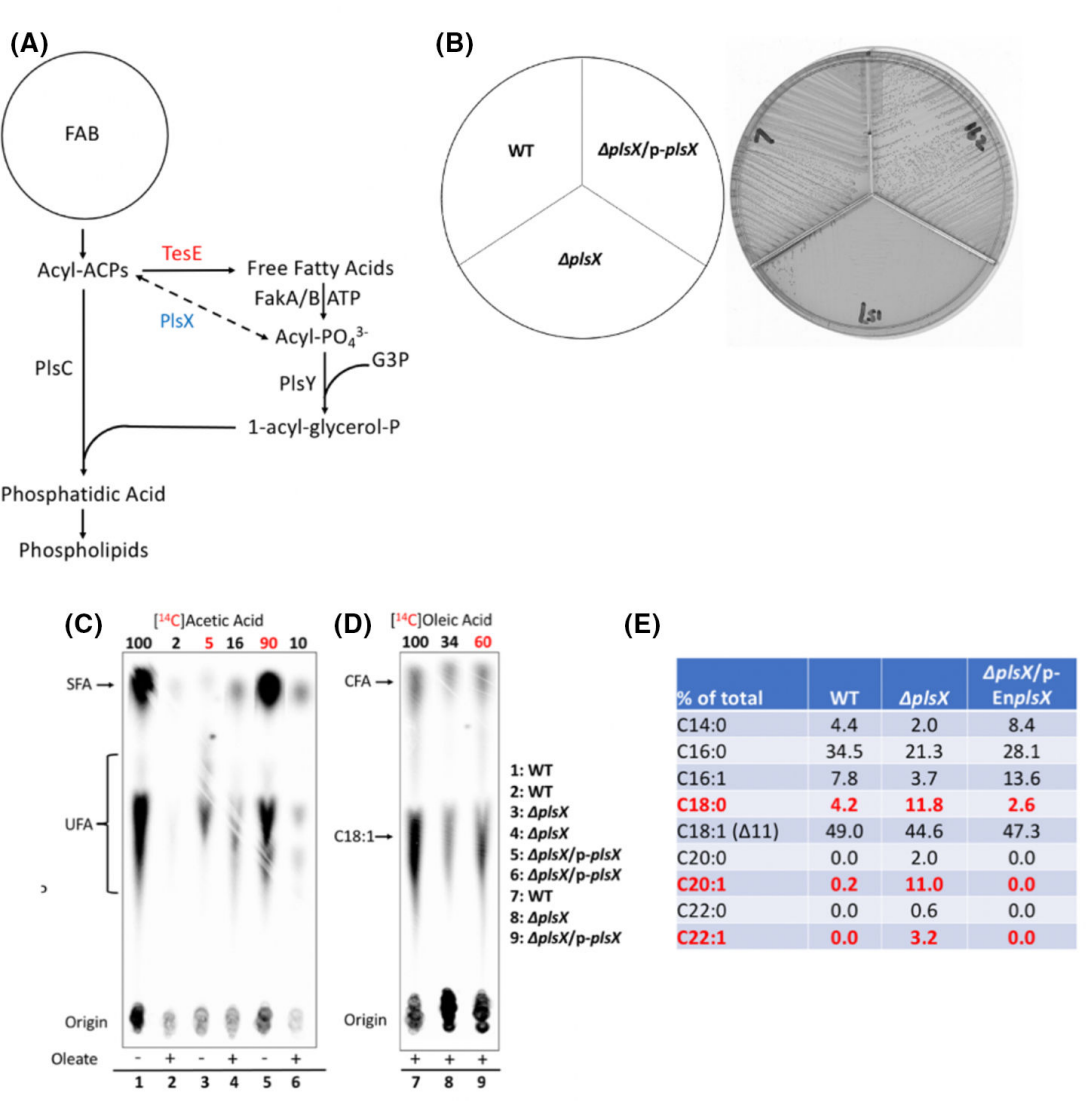
The role of PlsX in the phospholipid synthesis pathway of *E. faecalis* and the phenotypes of *E. faecalis* ∆*plsX* strains. (**A)** The *E. faecalis* phospholipid synthesis pathway. A similar pathway is present in *S. pneumoniae*. (**B)** Growth of the *E. faecalis* wild-type (WT), ∆*plsX*, and complemented ∆*plsX* (/p-*plsX*) strains on M17 agarose. (**C)** *De novo* phospholipid acyl chain synthesis of the wild-type, ∆*plsX,* and complemented ∆*plsX* strains assayed by [1-^14^C]acetate labeling. The numbers above the lanes are the radioactive label incorporation relative to the value (100) for the wild-type strain. The red numbers are to focus the reader on the relevant data above (**D)**. Incorporation of [1-^14^C]oleic acid by wild-type, ∆*plsX,* and complemented strains. The numbers above the lanes are the radioactive label incorporations relative to the value for the wild-type strain. The red numbers are to focus the reader on the relevant data. The thin layer chromatography lanes are identified on the figure. (**E)** Gas chromatography–mass spectrometry (GC-MS) analysis of the phospholipid acyl chains of the *E. faecalis* wild-type, ∆*plsX,* and complemented ∆*plsX/*p-*EnplsX* strains. A repeat experiment of that in 1C showed similar *de novo* phospholipid acyl chain synthesis in the ∆*plsX*/p*-LlplsX* strain. A repeat experiment of that in panel D showed similar incorporation of [1-^14^C]oleic acid in the ∆*plsX*/*pLlplsX* strain. For panel E, the results for each strain are from independent triplicate cultures. Abbreviations: SFA, saturated fatty acid; UFA, unsaturated fatty acid. *LlplsX, Lactococcus lactis plsX*

PlsX is a peripheral membrane acyltransferase having an α/β/α sandwich structure that functions as a dimer (5 - 7). Loss of PlsX leads to fatty acid auxotrophy in *S. aureus,* whereas this loss has no effect on the growth of *S. pneumoniae* (4, 8). Growth of the *S. pneumoniae* ∆*plsX* strain is due to the presence of the TesS thioesterase that cleaves acyl-ACPs produced by *de novo* fatty acid biosynthesis (8). The resulting free fatty acids are activated by the FakA/B system to form the acyl-PO_4_ required for G3P acylation at the *sn*1-position to initiate phospholipid synthesis (Fig. 1A) (2).

*E. faecalis* is an opportunistic pathogen with the high level of antibiotic resistance and can cause hospital-acquired diseases (9). *E. faecalis* inhabits the gastrointestinal tracts of humans and other animals as a facultative anaerobe (9). *E. faecalis* synthesizes membrane phospholipids from either *de novo* synthesized fatty acids or from exogenous free fatty acids and PlsX plays a key role in both processes (10 - 12). Like *S. pneumoniae*, *E. faecalis* has clustered *fab* genes and its *plsX* gene is located adjacent to the *acpB* gene encoding an auxiliary ACP involved in the regulation of fatty acid synthesis (10 - 12). However, the *E. faecalis* ∆*plsX* strain differs from that of *S. pneumoniae* in that the *E. faecalis* ∆*plsX* strain shows extremely weak growth and is deficient in phospholipid acyl chain synthesis, whereas the *S. pneumoniae* ∆*plsX* strain grows normally (8).

We report that the *E. faecalis* ∆*plsX* strain is a fatty acid auxotroph and growth was restored by supplementation with appropriate exogenous fatty acids. Although *E. faecalis* encodes TesE, a functional thioesterase, this enzyme does not restore growth as was reported for the *S. pneumoniae* ∆*plsX* strain (8). However, on high-level overexpression of TesE, growth was restored. A ∆*plsX* ∆*fabT* strain lacking the FabT repressor had a growth phenotype similar to that of the ∆*plsX* strain, although it showed enhanced *de novo* phospholipid acyl chain synthesis compared to the ∆*plsX* strain. We identified a ∆*plsX* suppressor strain that allowed wild-type growth by giving increased synthesis of saturated fatty acyl-ACP species which following PlsX-catalyzed conversion to acyl-PO_4_ initiate phospholipid synthesis by acylation of the *sn*1-position of G3P. Overexpression of an enoyl-ACP reductase had a similar effect. TesE has a marked preference for the cleavage of unsaturated acyl-ACPs, and overproduction of saturated acyl-ACPs overcomes this preference.

## MATERIALS AND METHODS

### Materials

The 15-methyl palmitic acid was purchased from Cayman Chemicals and all the other fatty acids, antibiotics, phospholipase A2, ortho-nitrophenyl-β-galactoside, and agmatine sulfate were purchased from Sigma-Aldrich. The media were purchased from Fisher Scientific. The DNA polymerase, restriction endonuclease, and T4 ligase were purchased from New England Biolabs. Sodium[1-^14^C]acetate (specific activity, 57.0 mCi/mmol) and [1-^14^C]stearic acid (specific activity, 53.0 mCi/mmol) were provided by Moravek, Inc, and the [1-^14^C]oleic acid (specific activity, 55 mCi/mmol) was purchased from American Radiolabeled Chemicals. Ni-NTA resin was from Invitrogen, and the DNA purification kits were from Qiagen. Silver nitrate silica gel thin layer plates were purchased from Analtech and M17 Broth was from Becton Dickenson. All the other reagents were of the highest available quality. Oligonucleotide primers were synthesized by Integrated DNA Technologies, and DNA sequencing was performed by ACGT, Inc.

### Bacterial strains, plasmids, and incubation

The bacterial strains and plasmids used in this study are listed in Table S1, and the primers used for this study are listed in Table S2. *E. coli* cultures were incubated at 37℃ in Luria-Bertani medium (tryptone, 10 g/L; yeast extract, 5 g/L; NaCl, 10 g/L), whereas *E. faecalis* cultures were grown at 37℃ in M17 medium (BD DIFCO) or in AC medium (tryptone, 10 g/L; yeast extract, 10 g/L; K_2_HPO_4_, 5 g/L; glucose, 1 g/L). Antibiotics were added at the following concentrations (in milligram per liter): sodium ampicillin, 100 for *E. coli*; kanamycin sulfate 50 for *E. coli*; chloramphenicol 30 for *E. coli,* and 5 for *E. faecalis*; erythromycin at 250 for *E. coli* and 5 for *E. faecalis*. Fatty acids were added at 0.1 mM unless otherwise stipulated.

### Construction of the *E. faecalis* ∆*plsX* ∆*fabT* strain

The construction of *E. faecalis* ∆*plsX* ∆*fabT* strain used plasmid pQZ149 containing the *plsX* gene knockout cassette described in the previous work (11, 12). *E. faecalis* ∆*fabT* cells transformed with the plasmid were selected on AC medium agar plates with 5 mg/L erythromycin and 100 mg/L 5-bromo-4-chloro-3-indolyl-β-D-galactopyranoside (X-Gal) at 30℃. A blue colony was selected and streaked on AC agar plates with the same components described above at 42℃ to verify plasmid integration into the genome. The blue colony was incubated in AC liquid medium in the presence of 0.1 mM palmitate at 30℃ for 4 hours, and the culture was shifted to 42℃ overnight. This process was repeated several times, and the culture was diluted and spread on AC agar plates with 0.1 mM palmitate and 100 mg/L X-Gal at 42℃. The white colonies were picked for colony PCR to verify the deletion of the *plsX* gene.

### *E. faecalis* thioesterase assay

The plasmid for expressing *E. faecalis* thioesterase was constructed by inserting its coding region amplified by primer set EftesE *NdeI F* and EftesE *EcoRI* R into the vector pET28b NdeI and EcoRI sites. To express *E. faecalis* thioesterase, *E. coli* Rosetta cells transformed with the expression plasmid above were incubated at 37℃ to optical density at 600 nm (OD_600_) of 0.6 and then induced with 1 mM isopropyl β-D-1-thiogalactopyranoside for 4 hours. The cells were harvested, washed with phosphate-buffered saline (PBS), resuspended with lysis buffer containing 50 mM sodium phosphate (pH 8.0), 0.3 M NaCl, 10 mM imidazole, and 1 mM dithiothreitol (DTT) and then lysed with a French Pressure Cell. The supernatant after centrifugation was loaded onto the Ni-NTA column, and the protein was eluted with 0.25 M imidazole, dialyzed, and stored with 20% glycerol at −80℃. To test the function of purified *E. faecalis* thioesterase *in vitro*, various species of *E. faecalis* acyl-ACPs were prepared as described in the previous work (11 - 13) and then mixed with the purified *E. faecalis* thioesterase in buffer containing 50 mM Tris-HCl (pH 8.0). The reaction was incubated at 37℃ for 1 hour, and the products were analyzed by conformation-sensitive 2 M urea–18% PAGE.

### Growth measurements of *E. faecalis* strains

The cultures were started at OD_600_ of 0.01 in M17 medium at 37℃ with or without the presence of a single exogenous fatty acid. The OD_600_ values of the culture were measured every 2 hours in triplicate for 8 hours.

### Construction of *E. faecalis plsX*, *fabK*, *fabI*, and *tesE* overexpression plasmids

The construction of *E. faecalis plsX* overexpression plasmid was slightly modified from that described previously (12), whereas the *plsX* gene was ligated to the pQZ28 vector derived from pZL277 vector by replacing the original chloramphenicol resistance gene with the erythromycin resistance gene from the pBVGh vector (12, 14, 15).

To construct the *E. faecalis fabK* overexpressing plasmid, the *fabK* gene was amplified from genomic DNA using primer set EffabK NcoI *F* and EffabK EcoRI R and then inserted into the NcoI and EcoRI sites of vector pQZ28 (12, 14, 15). For construction of the *fabI* overexpression plasmid the *fabI* gene was amplified using primers EffabI NcoI and EffabI EcoRI and inserted into vector pQZ28 as above.

To construct the *E. faecalis tesE* overexpressing plasmid, the *tesE* coding sequence together with its promoter was amplified using primer set EftesE promoter BamHI *F* and EftesE SmaI R and the produced fragment was inserted into the BamHI and SmaI sites of low-copy-number shuttle vector pTRKL2. To construct the *E. faecalis tesE* controllable overexpression plasmid, an agmatine-inducible system was used (10, 16). The fragment containing the promoter of putrescine transcarbamylase (*aguB*) and the regulator gene *aguR* was amplified using primer set EfaguR *EcoRI* R and EfaguB promoter R whereas the *tesE* coding region was amplified using primer set EftesE *F* and EftesE *NcoI* R. The two fragments synthesized above were combined through overlap PCR using primer set EfaguR *EcoRI* R and EntesE *NcoIR* and the resulting fragment was inserted into the NcoI and EcoRI sites of pQZ28 vector.

### β-galactosidase assays

The construction of reporter plasmids expressing *E. coli* β-galactosidase from *E. faecalis fabT* promoter, *fabI* promoter, or *fabO* promoter irrespectively has been described in the previous work (12). To construct a reporter plasmid expressing β-galactosidase from the *E. faecalis* thioesterase promoter, the promoter and the first 35 bp of *E. faecalis tesE* coding regions (−274 to +35) relative to the *tesE* gene GTG initiation codon were amplified using primer set EftesE promoter plus 35 *PstI F* and EftesE promoter plus 35 SalI R and inserted into PstI and SalI sites at the 5′ end of a promoterless *lacZ* gene on plasmid pBHK322 constructed in the previous work (12).

To assay β-galactosidase activity, *E. faecalis* strains transformed with *lacZ* reporter plasmids above were incubated to mid-log phase at 37℃, harvested through centrifugation, washed by PBS, resuspended in Z buffer, lysed with chloroform and sodium dodecyl sulfate, and assayed for β-galactosidase activity. The data were collected in triplicate.

### Thin layer chromatography (TLC) analysis of radioactive-labeled fatty acid methyl esters from bacterial phospholipids

To test *de novo* acyl chain biosynthesis, 5 mL of *E. faecalis* cultures was inoculated at OD_600_ of 0.1 in AC medium and incubated at 37℃ for 6 hours in the presence of 1 mCi/L sodium [1-^14^C]acetate with or without 0.1 mM of a single exogenous fatty acid. The cells were lysed with methanol–chloroform (2:1) solution, and the phospholipids were extracted by chloroform and then dried under nitrogen. The fatty acyl groups were methylated by 25% (wt/vol) sodium methoxide, extracted by hexanes, and processed for TLC analysis on Analtech silica gel containing 20% silver nitrate in toluene at −20°C. The TLC plates containing the [^14^C]-labeled fatty acid methyl esters were exposed and quantitated by phosphorimager analysis on a GE Typhoon FLA700 Scanner and the data were analyzed by ImageQuant TL software.

To test the incorporation of unsaturated fatty acids, *E. faecalis* strains were started at OD_600_ of 0.1 in 5 mL of AC medium containing 0.1 µCi/mL [1-^14^C]oleic acid with 0.1 mM non-radioactive oleate and cultured at 37℃ for 6 hours. The cells were washed with phosphate-buffered saline and the phospholipids were extracted, methylated, and analyzed as described above.

To test the incorporation of saturated fatty acids, *E. faecalis* strains were started at OD_600_ of 0.1 in AC medium containing 0.1 µCi/mL [1-^14^C]stearic acid plus 0.1 mM non-radioactive palmitate, cultured at 37℃ for 6 hours, and processed for TLC analysis as described above.

### Gas chromatography–mass spectrometry analysis of fatty acid components of cell membrane

*E. faecalis* strains were inoculated at an OD_600_ of 0.1 in AC medium with or without the presence of 0.1 mM of a single fatty acid and cultured at 37℃ for 6 hours. The conversion of phospholipids to fatty acid methyl esters was the same method used for TLC analysis above, and the extracted products were sent for gas chromatography–mass spectrometry analysis.

### Phospholipase A2 (PLA_2_) treatment for the positional analysis of fatty acids of bacterial phospholipids

The process to determine the positional analysis of the acyl chains of phospholipids has been described previously (17). The extracted phospholipids were hydrated at 25℃ for 20 min with 0.5-mL reaction buffer containing 0.1 mM Tris-HCl (pH 7.5), 10 mM CaCl_2_, and 10 mM MgCl_2_ followed by sonication at 4℃. Ten units of PLA2 (from bee venom, Sigma) dissolved in the same buffer was added and the reaction was incubated at 37℃ for 3 hours 0.5 mL of 0.5 M NaCl was added, and the reaction was terminated by adding 2 mL of methanol–chloroform (2:1). The organic phase was separated, collected, and 1-mL chloroform was added to complete the extraction. The digestion products were dried under nitrogen, and the lysophospholipid fraction was submitted for mass spectrometric analysis.

## RESULTS

### Loss of the *E. faecalis plsX* gene leads to growth deficiency and disruption of lipid metabolism

Unlike the *S. pneumoniae* ∆*plsX* strain (8) which grows normally, the *E. faecalis* ∆*plsX* strain showed only trace growth (very minute colonies) on M17 agarose plates which lack free fatty acids where the FA2-2 wild-type strain grew well (Fig. 1B). To test whether the growth deficiency of the ∆*plsX* strain was related to the inhibition of the synthesis of phospholipid acyl chains, we performed [1-^14^C]acetate labeling followed by argentation TLC, which showed a 20-fold decrease in phospholipid acyl chain synthesis (Fig. 1C). The ∆*plsX* strain also showed a three-fold decrease in [^14^C]oleic acid incorporation relative to the wild-type strain (Fig. 1D). Moreover, the ∆*plsX* strain synthesized dramatically increased levels of abnormally long-chain phospholipid acyl chains. Both saturated (C18:0) and unsaturated (C20:1 and C22:1) species were found although these are only trace components in the phospholipids of the wild-type strain (Fig. 1E). These abnormally long chains indicate reduced rates of transfer of acyl chains to G3P (or 1-acyl G3P) such that acyl-ACPs undergo additional cycles of elongation. This phenomenon was first seen in *E. coli* cultures starved for G3P (18). All ∆*plsX* phenotypes were abolished on the introduction of a plasmid-borne *plsX* gene into the ∆*plsX* strain (Fig. 1B through E). These data indicated that the growth defect of the *E. faecalis* ∆*plsX* strain was likely caused by poor acylation of G3P, which resulted in deficient phospholipid synthesis.

Note, that we have measured fatty acid synthesis indirectly by analysis of the phospholipid acyl chains. Although the acyl chains are made by the fatty acid synthesis pathway, we analyze the chains after they become phospholipid acyl groups. Hence, decreased incorporation of [1-^14^C]acetate can be due to the decreased synthesis *per se* or decreased transfer of acyl chains to the G3P phospholipid backbone or both. For example, when position *sn*1 of G3P is not acylated, position *sn*2 cannot be acylated.

### Exogenous fatty acids restore the growth of the *E. faecalis* ∆*plsX* strain

**Fig 2:**
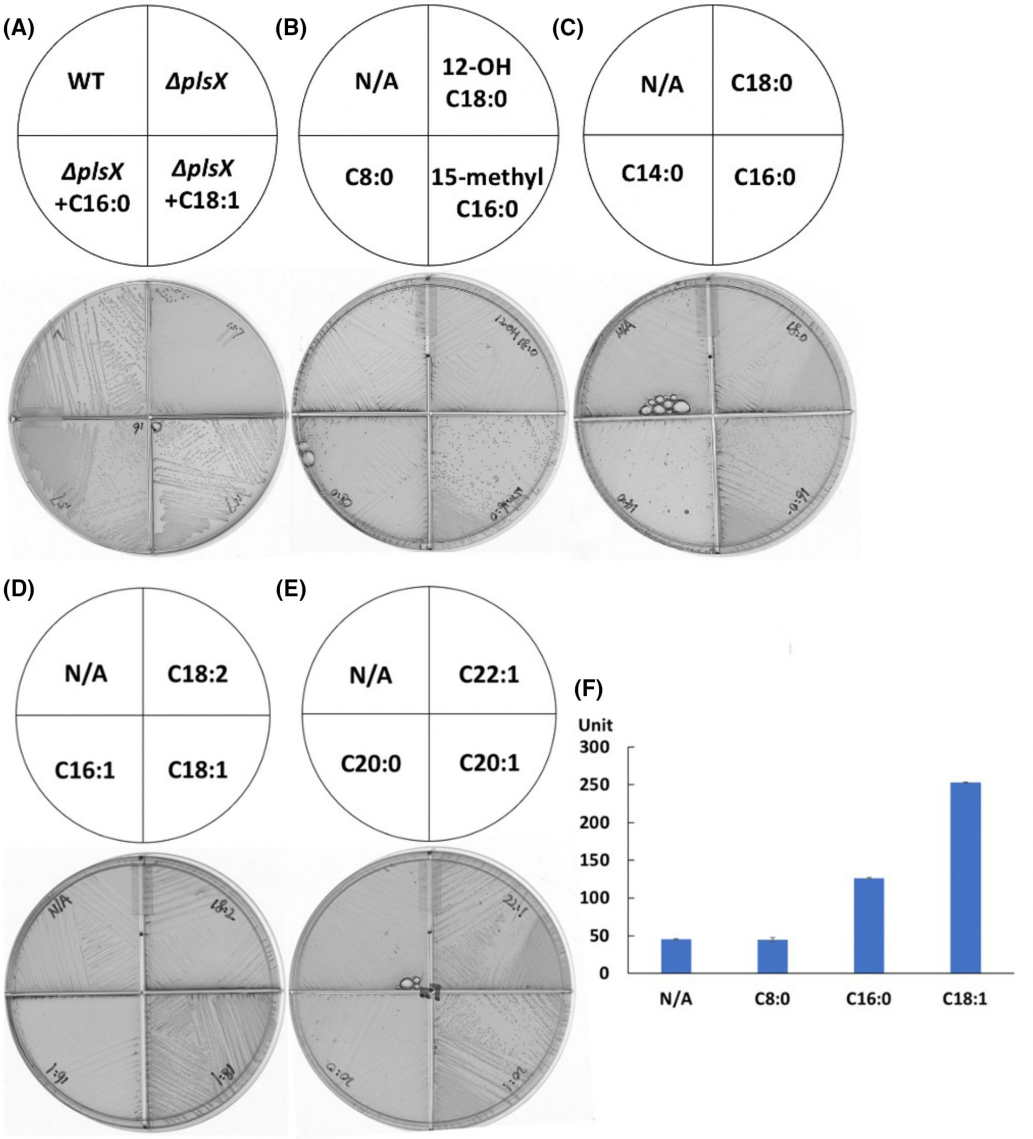
Exogenous fatty acids support the growth of *E. faecalis* ∆*plsX* strain in a specific manner. (**A–E)** Growth of the ∆*plsX* strain in the presence of various fatty acid species. (**F)** Expression of β-galactosidase from the *fabT* promoter in the ∆*plsX* strain grown in the presence of octanoic acid (C8:0), palmitic acid (C16:0), or oleic acid (C18:1). In the panel F, the *lacZ* expression from the *fabT* promoter in the ∆*plsX* strain in the presence of C16:0 or C18:1 was repeated with similar results, whereas that in the presence of C8:0 was from independent triplicate cultures. N/A denotes no additions.

To test the effects of various species of exogenous fatty acids on the growth of the *E. faecalis* ∆*plsX* strain, the strain was streaked on M17 agarose plates containing a single exogenous fatty acid at 0.1 mM. Supplementation with palmitic acid (C16:0) or oleic acid (C18:1, *cis*-9) restored the growth of the ∆*plsX* strain (Fig. 2A). Stearic acid (C18:0) failed to support growth on M17 agarose plates but supported good growth in M17 liquid medium (Fig. S1). A possible explanation is the agarose trapping of micelles of the very poorly soluble stearic acid. Growth could also be restored by 15-methyl palmitic acid (15-methyl C16:0) which can be distinguished by mass from endogenously synthesized palmitic acid (Fig. 2B). However, the short-chain fatty acid, octanoic acid (C8:0), and the hydroxy acid 12-hydroxyoctadecanoic acid (12-OH C18:0) failed to allow growth of the ∆*plsX* strain (Fig. 2B). Growth of the *E. faecalis* ∆*plsX* strain was not supported by myristic acid (C14:0), a minor component of the acyl chains of the wild-type strain phospholipids (Fig. 2C and Fig. 1D). Supplementation with palmitoleic acid (*cis*-9 C16:1) or linoleic acid (C18:2) failed to improve the growth of the ∆*plsX* strain (Fig. 2D), which could be caused by toxicity of these acids. In addition, supplementation with eicosenoic acid (*cis*-11 C20:1) or erucic acid (*cis*-13 C22:1) gave increased growth of the ∆*plsX* strain (Fig. 2E). To test the effects of exogenous fatty acids on the expression of *fab* genes in the ∆*plsX* strain, we measured *lacZ* gene expression driven by the *fabT* promoter (12). Relative to the ∆*plsX* strain incubated without exogenous fatty acids, *lacZ* expression was strongly increased in cells incubated with palmitic acid (threefold increase) or oleic acid (sixfold increase), whereas no change was observed in cultures grown with octanoic acid (Fig. 2F).

### Growth of the ∆*plsX* strain is restored by high-level expression of the TesE thioesterase

One possible explanation for the growth deficiency of the *E. faecalis* ∆*plsX* strain might be the lack of a functional acyl-ACP thioesterase which would block the release of free fatty acids from *de novo* synthesized acyl-ACPs. The lack of free fatty acids would preclude the production of acyl-PO_4_ by the fatty acid kinase system. Absence of acyl-PO_4_ for PlsY acylation of G3P position *sn*1 would block the initiation of phospholipid synthesis (Fig. 1A). Note that exogenous fatty acids bypass the need for a thioesterase because these are directly converted to acyl-PO_4_ species (4).

**Fig 3:**
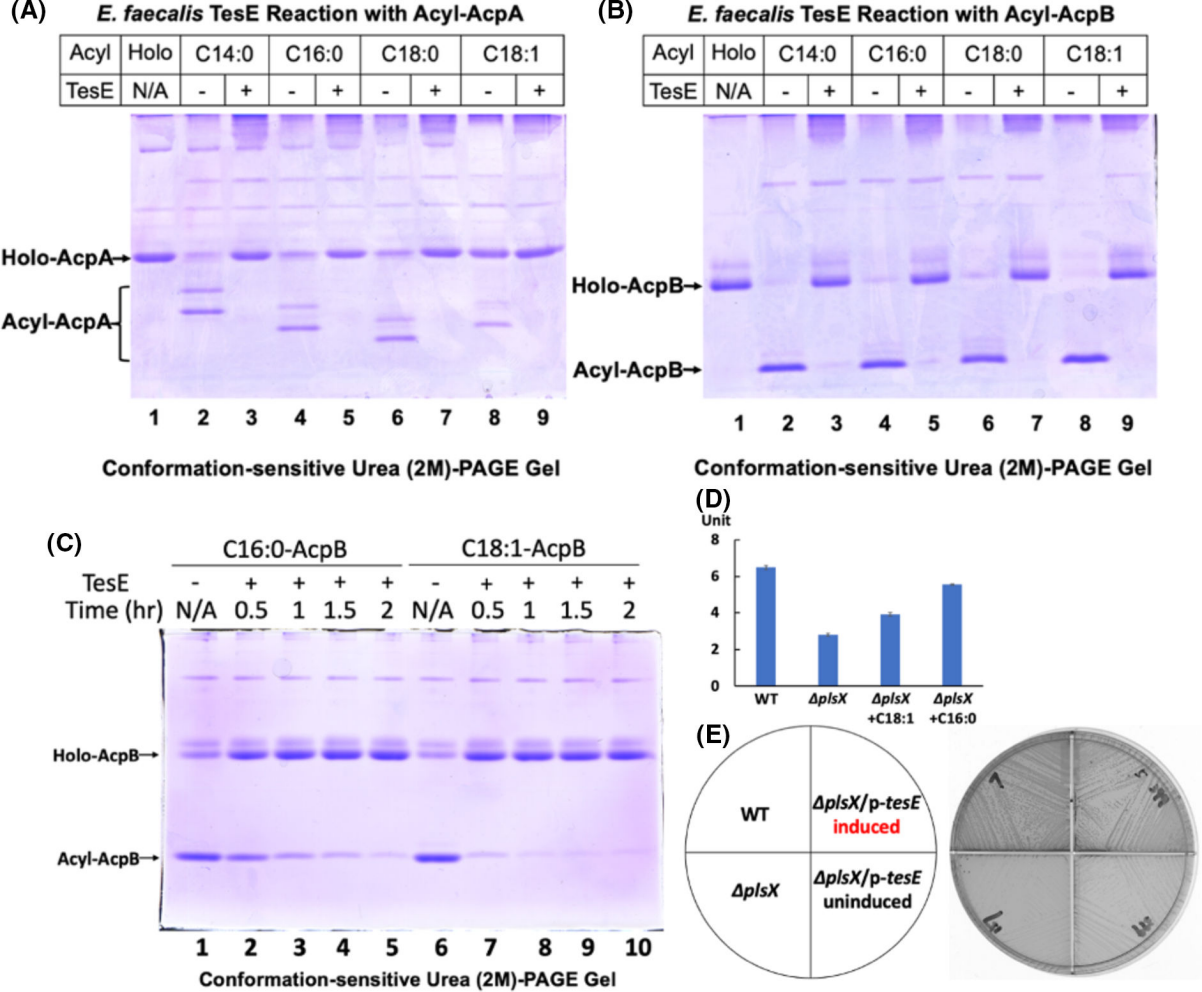
Demonstration of TesE thioesterase activity, expression of the gene, and the effects of TesE overexpression on growth of the ∆*plsX* strain. (**A and B)** Urea-PAGE gel electrophoresis of cleavage of *E. faecalis* acyl-ACPs by the purified TesE thioesterase. (**C)** Urea-PAGE gel electrophoresis of cleavage of *E. faecalis* palmitoyl-AcpB and oleoyl-AcpB by the purified TesE thioesterase. Synthesized palmitoyl-AcpB or oleoyl-AcpB was mixed with 25-nM *E. faecalis* thioesterase, and the reactions were incubated at 37°C for 0.5, 1, 1.5, or 2 hours. The reactions then were heated at 75°C to terminate the reactions, and the products were analyzed by conformation-sensitive PAGE. (**D)** Expression of β-galactosidase from the *tesE* promoter in the ∆*plsX* strain. (**E)** Growth of the ∆*plsX* strain overexpressing TesE on M17 agarose with or without the presence of 5 mM agmatine sulfate as inducer. In panel D, *lacZ* expression from the *tesE* promoter in the ∆*plsX* strain with the presence of C16:0 or C18:1 was from independent triplicate cultures. GC-MS analysis of the phospholipid acyl chains of the *E. faecalis* wild-type, ∆*plsX*, and ∆*plsX/*p-*tesE* strains either uninduced or induced is given in Table S3. N/A denotes no additions.

To test the thioesterase hypothesis, we identified an *E. faecalis* gene we call *tesE* (thioesterase *Enterococcus*) (locus tag EF_RS01825), which encodes a protein 39% identical to *S. pneumoniae* TesS. TesE was expressed and purified from *E. coli* and tested for the cleavage of various acyl species of both *E. faecalis* ACPs. Conformation-sensitive gel electrophoresis showed almost complete cleavage of acyl derivatives of both AcpA and AcpB to holo-ACP (Fig. 3A and B) indicating that *E. faecalis* TesE encodes a functional thioesterase. However, TesE is a weak thioesterase, probably even weaker than *S. pneumoniae* TesS, a reportedly weak enzyme (8). TesE shows a distinct preference for the cleavage of the unsaturated acyl-ACP, oleoyl-ACP, over the saturated acyl-ACP, palmitoyl-ACP (Fig. 3C), whereas *S. pneumoniae* TesS is reported to cleave these substrates equally well (8). A strong preference for oleoyl-ACP cleavage was seen in cell extracts (Fig. S2) as well as with the purified TesE. Expression of *E. faecalis tesE* gene assayed by a transcriptional fusion to β-galactosidase showed a 50% decrease in β-galactosidase activity in the ∆*plsX* strain compared to wild-type strain (Fig. 3D). However, overexpression of *E. faecalis* TesE strain from its native promoter on a low copy number plasmid failed to enhance the growth of the ∆*plsX* strain. We therefore used the regulated agmatine expression system (16) to allow the construction of a TesE expression plasmid. Exogenous agmatine controls the expression of agmatine degradation genes (16). A high copy plasmid (pQZ28) containing the *aguR* gene (which encodes a LuxR family transcriptional regulator of agmatine degradation) together with the promoter for the agmatine degradation genes was used to give inducible expression by agmatine addition. On induction with agmatine, the ∆*plsX* strain grew without fatty acid supplementation, whereas no growth was seen in the absence of induction (Fig. 3E and Fig. S3). Therefore, the growth deficiency of the ∆*plsX* strain is due to insufficient expression of the chromosomal *tesE* gene. Note that we made extensive attempts to delete *tesE* from the *E. faecalis* chromosome without success suggesting that it has an essential role in metabolism.

### *E. faecalis* ∆*plsX* ∆*fabT* strain has essentially the same growth phenotype as the *∆plsX* strain

**Fig 4:**
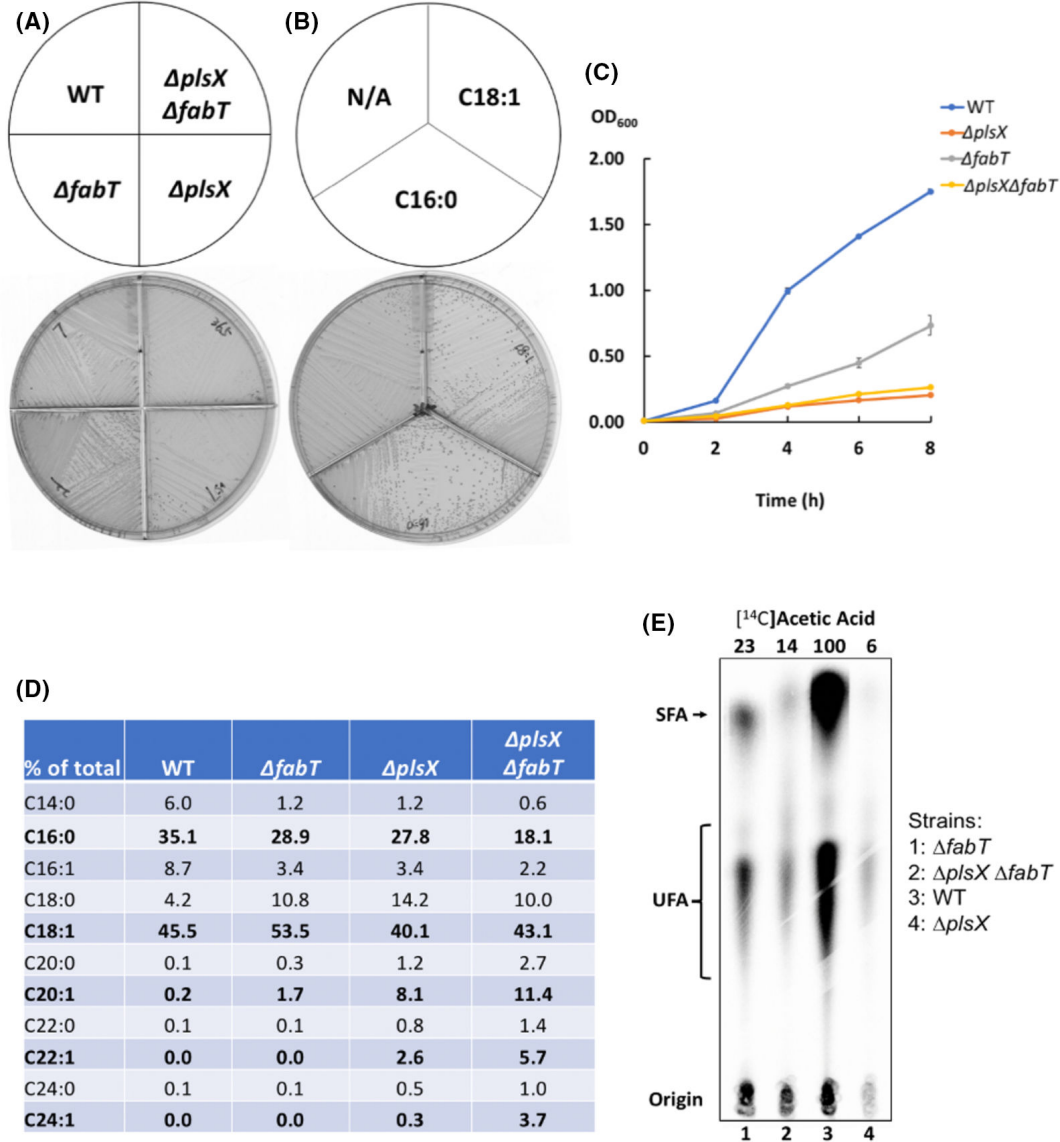
Deletion of *fabT* in the ∆*plsX* strain failed to correct the growth deficiency and abnormally long acyl chain synthesis of the ∆*plsX* strain. (**A and B)** Growth of the ∆*plsX* ∆*fabT* strain on M17 agarose either lacking (**A**) or containing exogenous fatty acids (**B**). (C) Growth curves of the wild-type, ∆*plsX*, ∆*fabT,* and ∆*plsX* ∆*fabT* strains in M17 medium. (**D)** GC-MS analysis of the acyl chains of the ∆*plsX*, ∆*fabT,* and ∆*plsX* ∆*fabT* strains. The relevant data are given in bold font. (**E)** *De novo* phospholipid acyl chain synthesis of wild-type, ∆*plsX*, ∆*fabT,* and ∆*plsX* ∆*fabT* strains assayed by [1-^14^C]acetate labeling. The relative labeling values are given at the top of the figure as in Fig. 1. In panel C, the growth curve for each strain was measured from independent triplicate cultures. In panel D, the result for each strain was from independent triplicate cultures. In panel E, a repeat experiment showed similar *de novo* phospholipid acyl chain synthesis in the ∆*plsX* ∆*fabT* strain. N/A denotes no additions.

The above data suggested that the growth deficiency of *E. faecalis* ∆*plsX* strain might result from a deficiency in *de novo* fatty acid biosynthesis caused by weak expression of *fab* genes. To test this premise, the ∆*plsX* ∆*fabT* strain was constructed to increase the expression of the *fab* genes (12). Lack of the FabT repressor increases the expression of the fatty acid synthesis genes by about threefold. However, the *E. faecalis* ∆*plsX* ∆*fabT* and ∆*plsX* strains showed similar growth-defective phenotypes (Fig. 4A through C). As expected, exogenous fatty acid supplementation restored the growth of the ∆*plsX* ∆*fabT* strain (Fig. 4B). Examination of the fatty acyl chain compositions of the phospholipids showed a higher proportion of the long-chain unsaturated fatty acyl chains C20:1, C22:1, and C24:1 in the ∆*plsX* ∆*fabT* strain (Fig. 4D). Labeling with [1-^14^C]acetate showed a two-fold increase in *de novo* phospholipid acyl chain fatty acid synthesis in the ∆*plsX* ∆*fabT* strain relative to the ∆*plsX* strain (Fig. 4E) as expected from increased *fab* gene expression. The increased levels of ≥C20-long unsaturated long-chain fatty acyl chains in the ∆*plsX* ∆*fabT* strain phospholipids result from increased elongation capacity. Indeed, the level of the C24 unsaturated acyl chain (presumably C24∆17) of the ∆*plsX* ∆*fabT* strain showed an increase of >10-fold relative to the ∆*plsX* strain (Fig. 4D). These data show that defective acylation of G3P rather than a lack of acyl chain synthesis causes the defective growth of the ∆*plsX* strain, and the wild-type level of TesE expression is too low to provide relief.

### Increased synthesis of saturated fatty acyl chains in the *E. faecalis ∆plsX* suppressor strain

**Fig 5:**
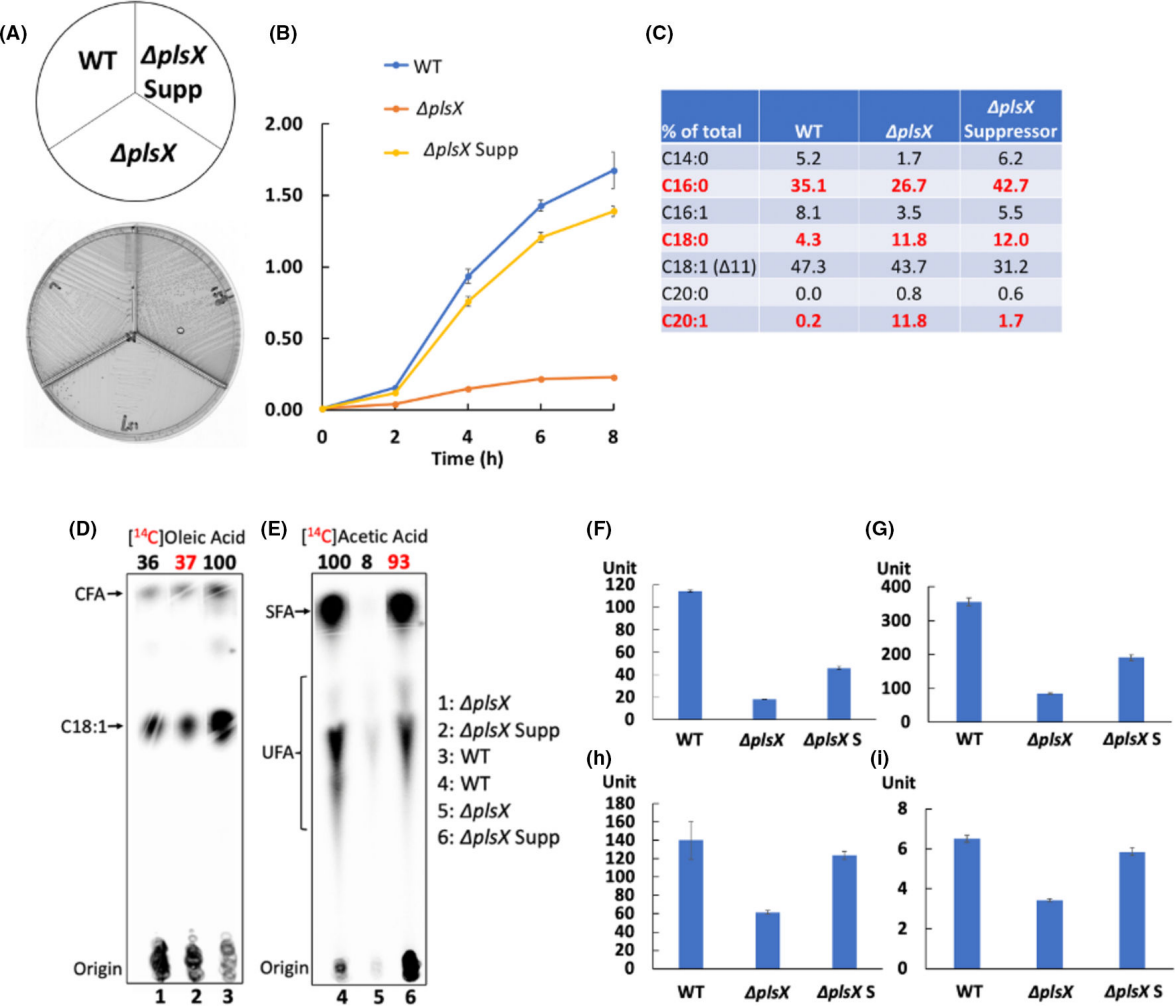
*E. faecalis* ∆*plsX* suppressor strain restored bacterial growth by enhancing saturated fatty acid synthesis. (**A)** Growth of the wild-type, ∆*plsX,* and ∆*plsX* suppressor strains on an M17 agarose plate. (**B)** Growth curves of the wild-type, ∆*plsX,* and ∆*plsX* suppressor strains in M17 medium. (**C)** GC-MS analysis of the acyl chain compositions of the wild-type, ∆*plsX*, and ∆*plsX* suppressor strains. (**D)** Incorporation of [1-^14^C]oleic acid by wild-type, ∆*plsX,* and ∆*plsX* suppressor strains. Inhibition of oleic acid incorporation in the ∆*plsX* and ∆*plsX* suppressor strains was also seen by GC-MS (Table S4). (**E)** *De novo* phospholipid acyl chain synthesis determined by [1-^14^C]acetate labeling of wild-type, ∆*plsX*, and ∆*plsX* suppressor strains. The isotopic compound incorporated is given above the autoradiograms. The numbers above the lanes are the radioactive label incorporations relative to the value for the wild-type strain (100). The red numbers are to focus the reader on the relevant data. **(F–H)** Expression of β-galactosidase from the *fabT* promoter (F), *fabI* promoter (G), and *fabO* promoter (H) in the ∆*plsX* suppressor strain (∆*plsX* S). **(I) **Expression of β-galactosidase from the *tesE* promoter in the ∆*plsX* suppressor strain. In panel B the growth curve for each strain was measured in independent triplicate cultures. In panel C, results for each strain were from independent triplicate cultures.In panel E, a repeat experiment showed similar *de novo* phospholipid acyl chain synthesis in the ∆*plsX* suppressor strain. In panel F, a similar higher expression of *lacZ* from *fabT* promoter in ∆*plsX* suppressor strain was detected in a separate experiment. In panels G and H, the *lacZ* expression from the *fabI* or *fabO* promoters in the ∆*plsX* suppressor strain was from independent triplicate cultures whereas that in the ∆*plsX* strain was repeated in an independent experiment. In panel I, *lacZ* expression from the *tesE* promoter for each strain was measured from independent triplicate cultures. For panel E, the lane of ∆*plsX* strain carrying *tesE* gene with its own promoter on the pTRKL2 low-copy vector at the right of ∆*plsX* suppressor was deleted since it was replaced with the strain overexpressing *tesE* gene from the agmatine-induced promoter on the pQZ28 high-copy vector in this study.

**Fig 6:**
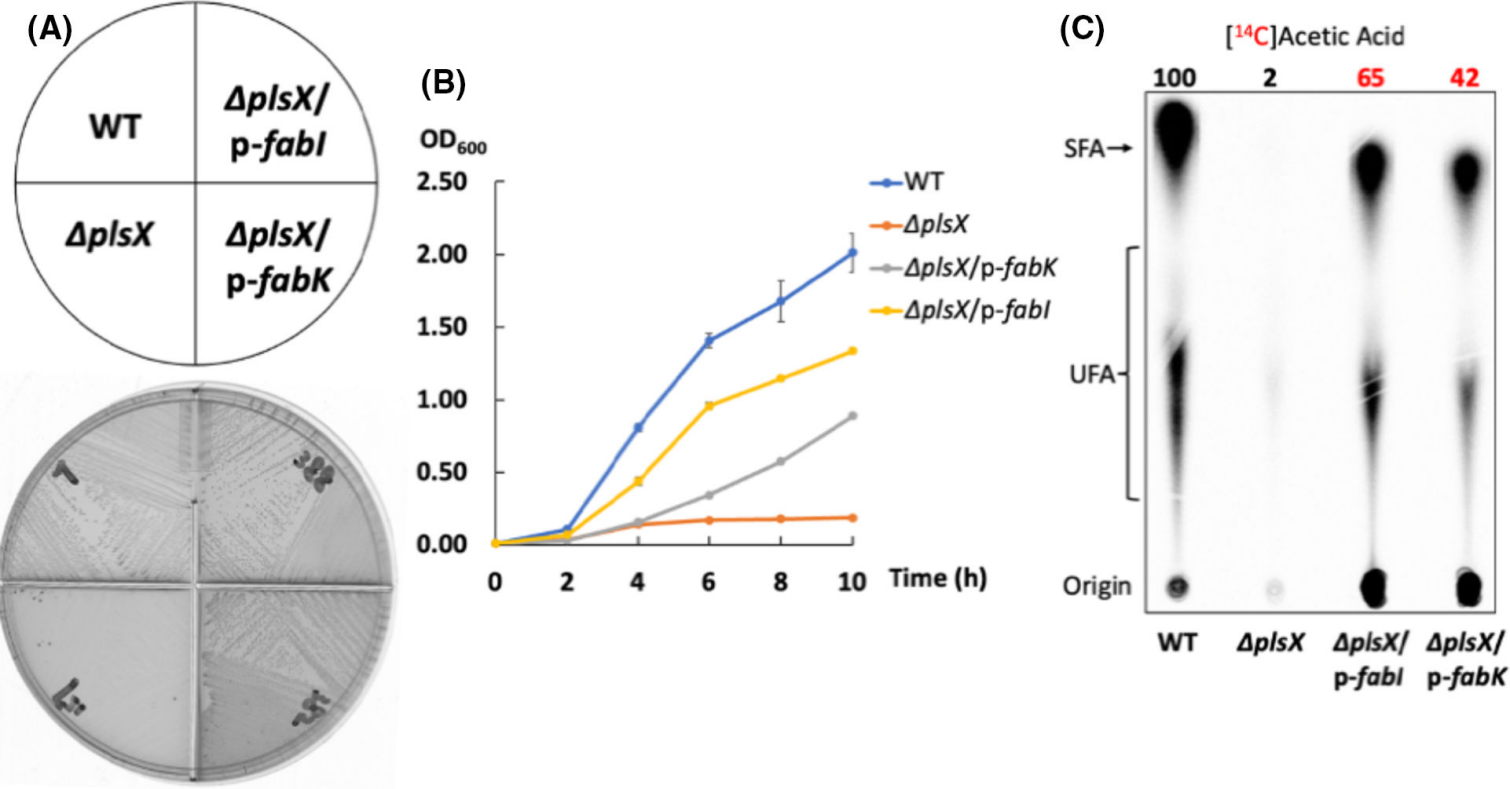
Overexpression of an *E. faecalis* enoyl-ACP reductase either FabI or FabK restores the growth of the ∆*plsX* strain. (**A)** Growth of *E. faecalis* ∆*plsX*/p-*fabK*, ∆*plsX*/p-*fabI*, wild-type (WT), and ∆*plsX* strains on M17 agarose. (**B)** Growth curves of wild-type, ∆*plsX*, ∆*plsX*/p-*fabK*, and ∆*plsX*/p-*fabI* strains. (**C)** *De novo* phospholipid acyl chain synthesis of wild-type, ∆*plsX*, ∆*plsX*/p-*fabI,* and ∆*plsX*/p-*fabK* strains assayed by [1-^14^C]acetate labeling. In panel B, the growth curve for each strain was measured from independent triplicate cultures. The numbers above the lanes are the radioactive label incorporation relative to the value for the wild-type strain (100). The red numbers are to focus the reader on the relevant data. In panel C, increased phospholipid acyl chain synthesis in the ∆*plsX*/p-*fabK* strain (88% of the wild-type strain) was detected in an independent experiment. GC-MS analysis of the phospholipid acyl chains of the *E. faecalis* wild-type, ∆*plsX,* and ∆*plsX/*p-*fabK* and ∆*plsX/*p-*fabI* strains is given in Table S4.

An *E. faecalis* ∆*plsX* suppressor colony isolated from a ∆*plsX* M17 agarose plate had a wild-type growth phenotype (Fig. 5 and B). Examination of the acyl chain components of the phospholipids showed a higher proportion of saturated acyl chains relative to the *E. faecalis* wild-type strain (Fig. 5C). When compared to the ∆*plsX* strain, the ∆*plsX* suppressor strain had a sixfold lower level of the C20:1 long-chain unsaturated acyl chain (Fig. 5C). However, the suppressor strain incorporated almost the same level of [1-^14^C]oleic acid as the ∆*plsX* strain (Fig. 5D). Labeling with [1-^14^C]acetate indicated a 12-fold increase in phospholipid acyl chain synthesis in the ∆*plsX* suppressor strain compared with the ∆*plsX* strain (Fig. 5E) compared to only a two- to threefold increase in the expression of the *fab* gene cluster as assayed by the *fab-lacZ* fusion (Fig. 5F-H). Moreover, expression of an *E. faecalis tesE-lacZ* fusion in the ∆*plsX* suppressor strain showed an about two-fold increase relative to the ∆*plsX* strain (Fig. 5I). High-throughput genomic sequencing (Oxford Nanopore confirmed by Illumina sequencing) of the ∆*plsX* suppressor strain identified a nonsense mutation (C1066 to *T*) in the *fabO* gene, which converted Gln356 to a termination codon resulting in a truncated FabO (β-ketoacyl-ACP synthase I) protein that lacked the last 56 residues. These residues are part of a β-sheet buried within the structure of the *S. pneumoniae* FabF (PDB 2ALM) and hence their loss is expected to destabilize and inactivate FabO. Note that unsaturated acyl chain synthesis is not blocked by the loss of FabO activity because the FabF 3-ketoacyl-ACP synthase II has weak FabO activity (10), which results in a strain having decreased synthesis of unsaturated acyl chains. Strikingly, the growth of the ∆*plsX* strain could be also recovered by overexpression of an *E. faecalis* enoyl-ACP reductase, either FabI or FabK (Fig. 6A and B). Overexpression of FabI or FabK results in greatly increased saturated acyl chain synthesis at the expense of unsaturated acyl chains (10, 11) and gave a 30-fold (FabI) or 20-fold (FabK) increase in *de novo* acyl chain synthesis compared to the ∆*plsX* strain lacking overexpression (Fig. 6C). However, the FabK overexpression strain grew more slowly than the FabI overexpression strain perhaps due to the alteration of cell membrane stability by excess saturated fatty acyl chains (Fig. 6B) (10).

### *E. faecalis* preferentially acylates the *sn*1-position of glycerophospholipids with saturated fatty acids

**Fig 7:**
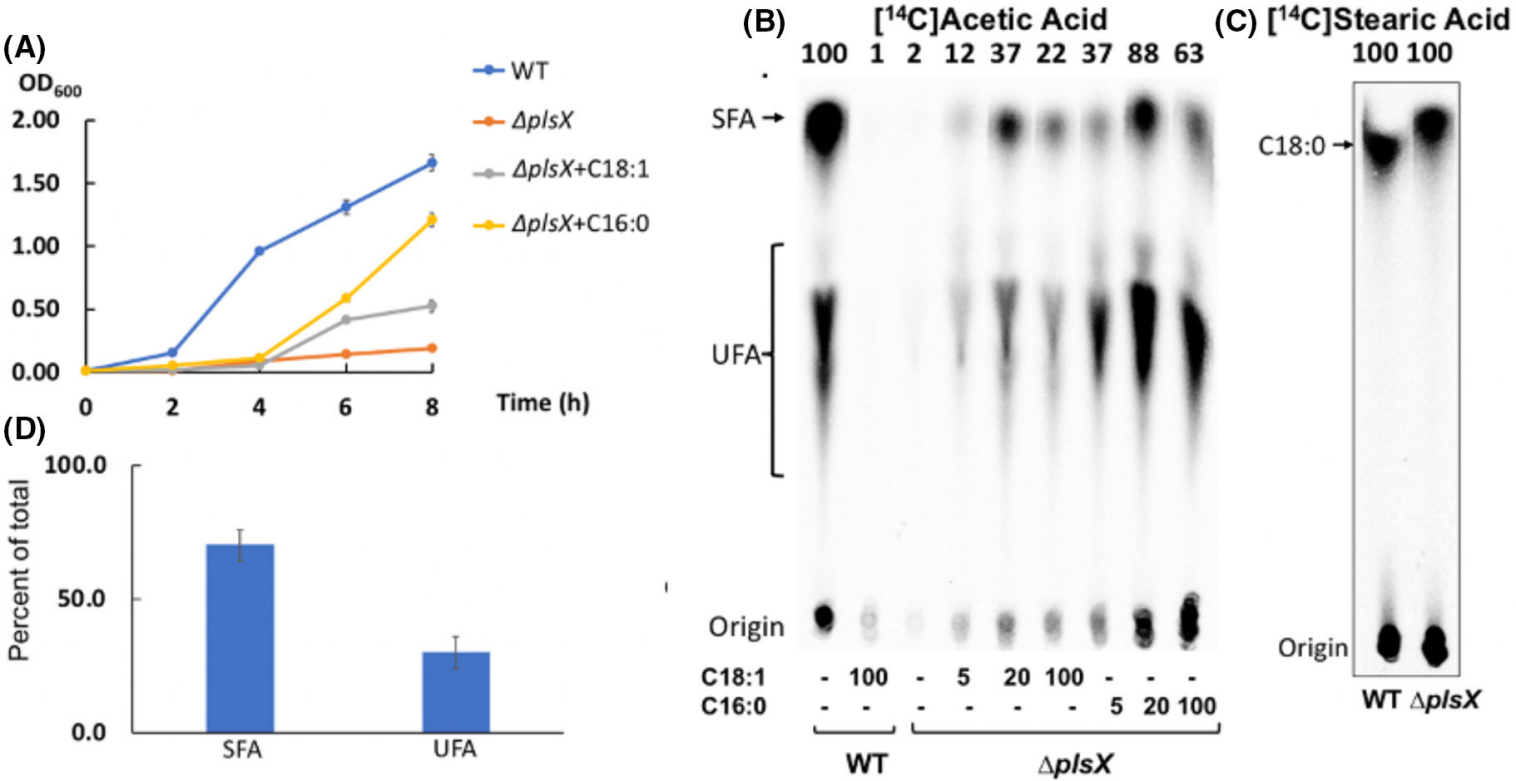
Saturated acyl chains are preferred for the acylation of the *sn*1-position of *E. faecalis* glycerophospholipids. (**A)** Growth curves of wild-type strain and ∆*plsX* strain incubated with or without either oleic acid (C18:1) or palmitic acid (C16:0). (**B)** *De novo* phospholipid fatty acyl chain synthesis of the wild-type and ∆*plsX* strains in the presence of various concentrations of either oleic acid or palmitic acid. The numbers above the lanes are the radioactive label incorporation relative to the value for the wild-type strain (100). (**C)** Incorporation of [1-^14^C]stearic acid by the wild-type and ∆*plsX* strains. The numbers above the lanes are the radioactive label incorporation relative to the value for the wild-type strain. (**D)** LC-MS analysis of the acyl chains of the *sn*1-position of glycerophospholipids of the *E. faecalis* wild-type strain. In panel A, the growth curve for each strain in each situation was measured from independent triplicate cultures. In panel C, similar [1-^14^C]stearic acid incorporation in the ∆*plsX* strain cultured with 0.1 mM non-radioactive stearic acid was detected in two other experiments. In panel D, the proportion of lysophospholipids for *E. faecalis* FA2-2 strain was measured in independent triplicate cultures.

To test the acyl chain composition of the *sn*1-position of *E. faecalis* phospholipids, the ∆*plsX* strain was grown with or without exogenous fatty acids (Fig. 7A). Growth of the ∆*plsX* strain increased when palmitic acid was present, indicating that saturated fatty acids in the G3P *sn*1-position enhance growth (Fig. 7A). Moreover [1-^14^C]acetate labeling showed that palmitic acid was more efficient than oleic acid in stimulating acyl chain synthesis in the ∆*plsX* strain (Fig. 7B). Unlike incorporation of [1-^14^C]oleic acid (Fig. 1D and Fig. 5D), labeling with [1-^14^C]stearic acid showed very similar incorporation into the cell membrane phospholipids of wild-type and ∆*plsX* strains (Fig. 7C). To determine the acyl chains esterified to the *sn*1-position of phospholipids in the *E. faecalis* wild-type phospholipids, phospholipase A2 (PLA_2_) was used to cleave the acyl chains at *sn*2-position and convert phosphatidylglycerol, the major phospholipid species, to 1-acylphosphatidylglycerol (17). Mass spectral analysis of the derived 1-acylphosphatidylglycerol indicated that about 70% of acyl chains were saturated, mainly C16:0 and C18:0, indicating that PlsY favors the incorporation of saturated acyl chains in *de novo* synthesis of glycerophospholipids (Fig. 7D).

## DISCUSSION

The growth of *E. faecalis* ∆*plsX* strain is extremely poorly. This is in marked contrast to the normal growth reported for *S. pneumoniae* ∆*plsX* strains (8). The poor growth of the *E. faecalis* ∆*plsX* strain results from an inability to efficiently acylate the G3P precursor of phospholipid synthesis, as shown by the dramatic increase in acyl chains of ≥C20. The growth defect of the *E. faecalis* ∆*plsX* strain can be overcome by the overproduction of saturated acyl-ACP species or by supplementation with exogenous fatty acids. The first clue to the role of increased saturated acyl-ACP species came from the ∆*plsX* suppressor strain that restored growth by expression of a truncated FabO protein. FabO encodes a 3-ketoacyl-ACP synthase I that elongates the *cis*-3-decenoyl-ACP required to initiate unsaturated fatty acid synthesis (10). However, the loss of FabO does not block unsaturated fatty acid synthesis in *E. faecalis* because the FabF 3-ketoacyl-ACP synthase II can weakly elongate *cis*-3-decenoyl-ACP and provide decreased, albeit sufficient, unsaturated fatty acid synthesis (10). The loss of FabO diverts nascent acyl-ACPs to saturated acyl-ACP species (Fig. 5C). Consistent with this result, the growth deficiency of the *E. faecalis* ∆*plsX* strain was also overcome by overexpression of the FabK or FabI enoyl-ACP reductases, which results in increased saturated acyl chain synthesis (Fig. 6A through C). On overexpression, these enoyl-ACP reductases intercept the *trans*-2 decenoyl-ACP intermediate of the FabN dehydrase/isomerase resulting in the blockage of unsaturated fatty acid synthesis (10).

Both FabO truncation and enoyl-ACP reductase overexpression bypass the loss of PlsX by increasing the synthesis of saturated acyl chains at the expense of unsaturated acyl chains. The most straightforward mechanism for bypass of loss of PlsX would be cleavage of the saturated acyl-ACPs by TesE to give fatty acids for the conversion to the acyl-PO_4_ species required for PlsY acylation of G3P position 1. TesE cleaves unsaturated acyl-ACPs much more readily than saturated acyl-ACPs (Fig. 3 and Fig. S2). However, the level of TesE activity produced by the chromosomal *tesE* gene is too low to provide sufficient saturated acyl-ACPs to allow normal growth of the ∆*plsX* strain. However, TesE overexpression increased the cleavage of saturated acyl-ACPs by outcompeting the unsaturated acyl-ACPs for access to TesE resulting in the production of saturated acyl chains. The saturated fatty acids are converted to acyl-PO_4_ that acylates the *sn*1-position of G3P and initiates phospholipid synthesis. This mechanism explains the rescue of growth of the ∆*plsX* strain by supplementation with palmitic acid, which is directly converted to palmitoyl-PO_4_.

Although this mechanism explains the data reported above, caveats remain. We have been unable to delete the *tesE* gene suggesting that it may have a physiological role other than acyl-ACP cleavage. Another complication is that the FakA fatty acid kinase activity requires a FakB fatty acid–binding protein. *E. faecalis* expresses four FakB proteins, whereas *S. pneumoniae* expresses three such proteins, each binding a defined fatty acid species (19). However, we have yet to detect fatty acid–binding specificity in any of the four *E. faecalis* FakB proteins (12). Moreover, the specificities of the *E. faecalis* G3P acyltransferases, PlsY and PlsC, have not been explored. Yet another complication is that the two *E. faecalis* ACPs have different biases in the PlsX reaction (11). AcpA favors the formation of acyl-phosphates, whereas AcpB favors the formation of acyl-ACPs. A further puzzle is the requirement for high levels of saturated acyl-ACPs to bypass the lack of PlsX. This is a puzzle because strains of *E. faecalis* totally blocked in fatty acid synthesis grow well with only oleic acid supplementation (15).

The saturated acyl-ACP requirement may be due to the loss of the substrate-channeling function of PlsX (7). Recent reports indicate that PlsX shuttles between the cytosol and membrane and that *in vivo* function of the enzyme requires membrane binding which is proposed due to PlsX-binding acyl-PO_4_ and presenting this substrate to the membrane-bound PlsY (6, 7). In the absence of PlsX, PlsY may show a strong preference for saturated versus unsaturated acyl-PO_4_ species. Further work will be required to understand these complex interactions.

It may seem surprising that the *E. faecalis* ∆*plsX* strain behaves differently from the *S. pneumoniae* ∆*plsX* strain since both species encode functional thioesterases that could provide fatty acids for acyl-PO_4_ synthesis. However, the *E. faecalis* thioesterase appears weaker than that of *S. pneumoniae*, poorly expresses and strongly favors the cleavage of unsaturated acyl-ACPs, whereas the *S. pneumoniae* thioesterase has no such bias (8). Moreover, TesE is not coregulated with the fatty acid synthesis genes (Fig. S4). In agreement with Parsons and coworkers (8), we suspect that the TesE and TesS activities are the side reactions of an esterase or an acyltransferase lacking its acyl acceptor.

## SUPPLEMENTAL MATERIAL

Supplemental materials msphere.00120-23-s0001.pdf Four tables and four figures.

ASM does not own the copyrights to Supplemental Material that may be linked to, or accessed through, an article. The authors have granted ASM a non-exclusive, world-wide license to publish the Supplemental Material files. Please contact the corresponding author directly for reuse.
