# Supplementary material for: Growth of Enterococcus faecalis ∆plsX strains is restored by increased saturated fatty acid synthesis
Source: mSphere. 2023 Jun 8;8(4):e00120-23. doi: 10.1128/msphere.00120-23 (PMC10449490; doi:10.1128/msphere.00120-23)
Supplement: Supplemental materials — Four tables and four figures. [file msphere.00120-23-s0001.pdf]

Supplement to:

Growth of *Enterococcus faecalis* Δ*plsX* Strains is Restored by Increased Saturated Fatty Acid Synthesis

Qi Zou, Huijuan Dong and John E. Cronan

Table S1 Strains and Plasmids

Strains and Plasmids	Description	Source
**Strains**		
E. coli Rossetta	*ompT hsdSB* (rB^{-} mB^{-}) *gal dcm* (DE3) pRARE (Cm^{r})	Novagen
E. faecalis FA2-2	Wild-Type	Lab Store
E. faecalis ZL116	Δ*fabT*	(1)
E. faecalis QZ157	Δ*plsX*	(2)
E. faecalis QZ158	Δ*plsX* suppressor (*fabO*)	This work
E. faecalis QZ162	Δ*plsX* with *plsX* expression plasmid	This work
E. faecalis QZ219	FA2-2 with *lacZ* expression from *fabT* promoter	(2)
E. faecalis QZ239	FA2-2 with *lacZ* expression from *fabI* promoter	(2)
E. faecalis QZ241	FA2-2 with *lacZ* expression from *fabO* promoter	(2)
E. faecalis QZ223	Δ*plsX* with *lacZ* expression from *fabT* promoter	(2)
E. faecalis QZ257	Δ*plsX* with *lacZ* expression from *fabI* promoter	This work
E. faecalis QZ258	Δ*plsX* with *lacZ* expression pfrom *fabO* promoter	This work
E. faecalis QZ252	Δ*plsX* suppressor with *lacZ* expression from *fabT* promoter	This work
E. faecalis QZ326	Δ*plsX* suppressor with *lacZ* expression from *fabI* promoter	This work
E. faecalis QZ327	Δ*plsX* suppressor with *lacZ* expression from *fabO* promoter	This work

E. faecalis QZ337	FA2-2 with *lacZ* expression from *tesE* promoter	This ork
E. faecalis QZ338	Δ*plsX* with *lacZ* expression from *tesE* promoter	This work
E. faecalis QZ339	Δ*plsX* suppressor with *lacZ* expression from *tesE* promoter	This work
E. faecalis QZ382	Δ*plsX* with *fabK* expression plasmid	This work
E. faecalis QZ388	Δ*plsX* with *fabI* expression plasmid	This work
E. faecalis QZ365	Δ*plsX* Δ*fabT*	This work
E. faecalis QZ399	Δ*plsX* with *tesE* expression plasmid	This work
**Plasmid**		
pBVGh	Temperature-sensitive β-galactosidase erythromycin-resistant gene modification vector	(3)
pZL277	Shuttle plasmid vector with a p32 promoter, *E. faecalis* expression	(1)
pQZ28	Shuttle plasmid vector with the p32 promoter modified from pZL277 by replacing the chloramphenicol-resistant gene with erythromycin-resistant gene, *E. faecalis* expression	This work
pQZ43	*E. faecalis plsX* in pQZ28	This work
pQZ149	*E. faecalis plsX* knockout cassette on vector pBVGh	(2)
pQZ18	*E. faecalis tesE* expression plasmid	This work
pQZ398	*E. faecalis tesE* in pQZ28 (agmatine-induced)	This work
pQZ379	*E. faecalis fabK* in pQZ28	This work
pQZ387	*E. faecalis fabI* in pQZ28	This work
pBHK322	promoterless *E. coli lacZ* on vector pTRKL2	(4)
pQZ332	*E. faecalis tesE* start region (-274 to +35) at 5'-end of *lacZ* in pBHK322	This work
pQZ214	*E. faecalis fabT* start region (-389 to +35) at 5'-end of *lacZ* in pBHK322	(2)
pQZ235	*E. faecalis fabI* start region (-297 to +35) at 5'-end of *lacZ* in pBHK322	(2)
pQZ238	*E. faecalis fabO* start region (-297 to +35) at 5'-end of *lacZ* in pBHK322	(2)

Table S2. Oligonucleotides primers used in the study

**Primers***	**Sequence 5'-3'**
Em SalI F	ACGCGTCGACGGTTCGTGTTCGTGCT
Em BamHI R	CGCGGATCCTTATTTCCTCCCGTTAA
EfplsX SmaI F	TCCCCCGGGATGAAAATTGCTGTAGATGC
EfplsX EcoRI R	CCGGAATTCTTACTCTGCTTTGCC
pZL277-EfplsX NcoI QC F	GAGGTGAACCATGCCCG
pZL277-EfplsX NcoI QC R	CGGGCATGGTTCACCTC
EftesE NdeI F	GGGTTTCATATGGTGGGAAAAAAACATA
EftesE EcoRI R	CCGGAATTCTTAGTTGGTCCAATCGAT
EfaguR EcoRI R	CCGGAATTCTTATCTCGCTAAATGTTCAGTT
EfaguB Promoter R	ATGTTTTTTTCCCATGATGTGTTCCTCCTAAAAGT
EftesE F	TAGGAGGAACACATCATGGGAAAAAAACATACAT CCT
EftesE NcoI R	CATGCCATGGTTAGTTGGTCCAATCGATATT
EftesE promoter plus 35 PstI F	AAAATTCTGCAGGAAAAAAACCTCCTAAAAAATTA TTTT
EftesE promoter plus 35 SalI R	ACGCGTCGACGCAACTTCATAAGAGGATGTAT
EffabK NcoI F	CATGCCATGGCTATGAAGTGTACTTATCTTAGAAC TAAAGGA
EffabK EcoRI R	CCGGAATTCTTAGCCCCAACGCTGAT
EffabI NcoI F	CATGCCATGGCTATGTTTTTACAAAATAAGAATGT CGT
EffabI EcoRI R	CCGGAATTCTTAAGTTAAGTGAACGCCTTT

* The primer sequences were based on the *E. faecalis* V583 genome. The underlined sequences indicate the restriction sites used in the study.

Table S2. GC-MS Analysis of the phospholipid acyl chains of *E. faecalis* wildtype, Δ*plsX* and Δ*plsX/p-tesE* uninduced and induced strains.

Proportion	WT	Δ*plsX*	Δ*plsX*/p-*tesE* Uninduced	Δ*plsX*/p-*tesE* Induced
C14:0	3.1	1.0	0.9	0.7
**C16:0**	**33.6**	**27.7**	**21.9**	**17.5**
C16:1	10.1	1.3	2.9	2.7
C18:0	6.4	17.8	13.0	9.4
**C18:1 (Δ11)**	**42.2**	**23.0**	**39.8**	**50.6**
C20:0	2.2	9.6	5.3	4.2
C20:1	0.1	5.3	7.3	7.9
C22:0	1.6	8.1	4.7	3.7
C22:1	0.0	0.9	1.0	0.8
C24:0	0.8	5.3	3.2	2.6

Table S3. GC-MS Analysis of Incorporation of exogenous C18:1 (*cis*-9) by the *E. faecalis* wildtype, Δ*plsX* and Δ*plsX* suppressor strains.

% of total	WT+C18:1	Δ*plsX*+C18:1	Δ*plsX* Supp+C18:1
C14:0	0.5	11.6	16.5
C16:0	5.7	15.6	19.1
C16:1	0.5	6.8	2.2
C18:0	2.7	1.7	2.1
**C18:1 (Δ9)**	**88.7**	**53.3**	**56.7**
C18:1 (Δ11)	1.8	11.1	3.3

Table S4. GC-MS Analysis of the phospholipid acyl chains of *E. faecalis* wildtype, Δ*plsX*. Δ*plsX*/p-*fabK* and Δ*plsX*/p-*fabI* strains.

**Proportion**	**WT**	***ΔplsX***	**Δ*plsX*/p-*fabK***	**Δ*plsX*/p-*fabI***
C14:0	2.9	0.8	3.0	2.4
**C16:0**	**29.1**	23.2	**42.0**	**37.9**
C16:1	7.7	1.4	2.6	2.9
**C18:0**	**6.5**	18.1	**15.6**	**13.7**
C18:1	45.0	22.1	25.0	34.3
C20:0	2.7	10.0	3.6	2.8
C20:1	0.2	6.3	1.8	1.7
C22:0	3.4	10.4	3.5	2.5
C24:0	2.5	7.7	2.7	1.8

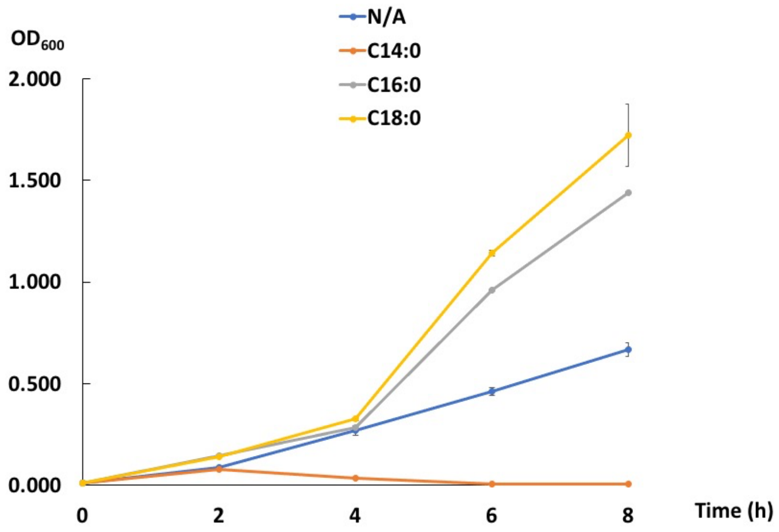

Fig. S1. Growth of the Δ*plsX* strain in M17 medium containing saturated fatty acids. The growth curves for the Δ*plsX* strain was measured from independent duplicate cultures. Similar restored growth of the *E. faecalis* Δ*plsX* strain in the presence of C18:0 was observed in an independent experiment.

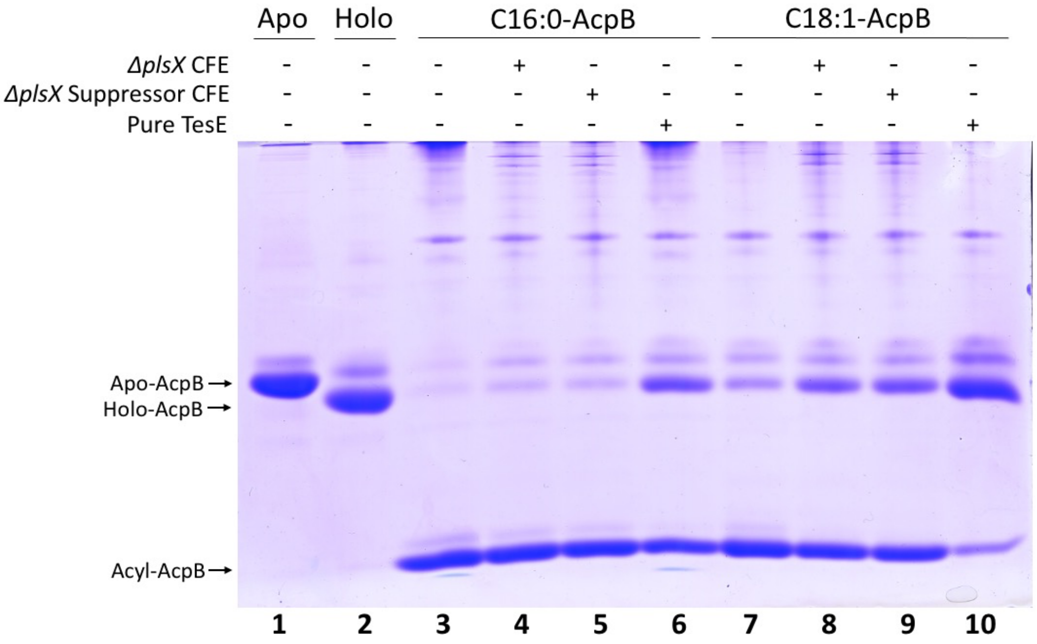

Fig. S2. Thioesterase activities versus saturated and unsaturated acyl-ACPs. A cell free extract (CFE) partially purified by ammonium sulfate precipitation was assayed. Cultures of the *E. faecalis* Δ*plsX* strain and the *fabO* suppressor strain were inoculated at OD_{600} 0.1 and grown to log phase. The harvested cells were resuspended with lysis buffer containing 0.1M sodium-phosphate (pH 7.0), 1 mM EDTA and 1 mM DTT and lysed by French Press treatment. Cell debris was precipitated through centrifugation and the supernatant was first treated with 40% followed by 85% ammonium sulfate (% of saturation). The pellets were resuspended with lysis buffer and dialyzed at 4°C overnight with buffer containing 0.1 M sodium phosphate (pH 7.0), 0.3 M NaCl, 1 mM EDTA, 1 mM DTT and 20% glycerol. The total protein concentration in produced cell-free extract was determined through Bradford Assay and normalized to 1 mg/ml palmitoyl-AcpB or oleoyl-AcpB were mixed with the cell-free extracts (2 μg protein) from the Δ*plsX* or ΔplsX suppressor strains and the reactions were incubated at 37°C for 2 h. The products were analyzed by conformation-sensitive 2 M urea-18% polyacrylamide gel electrophoresis.

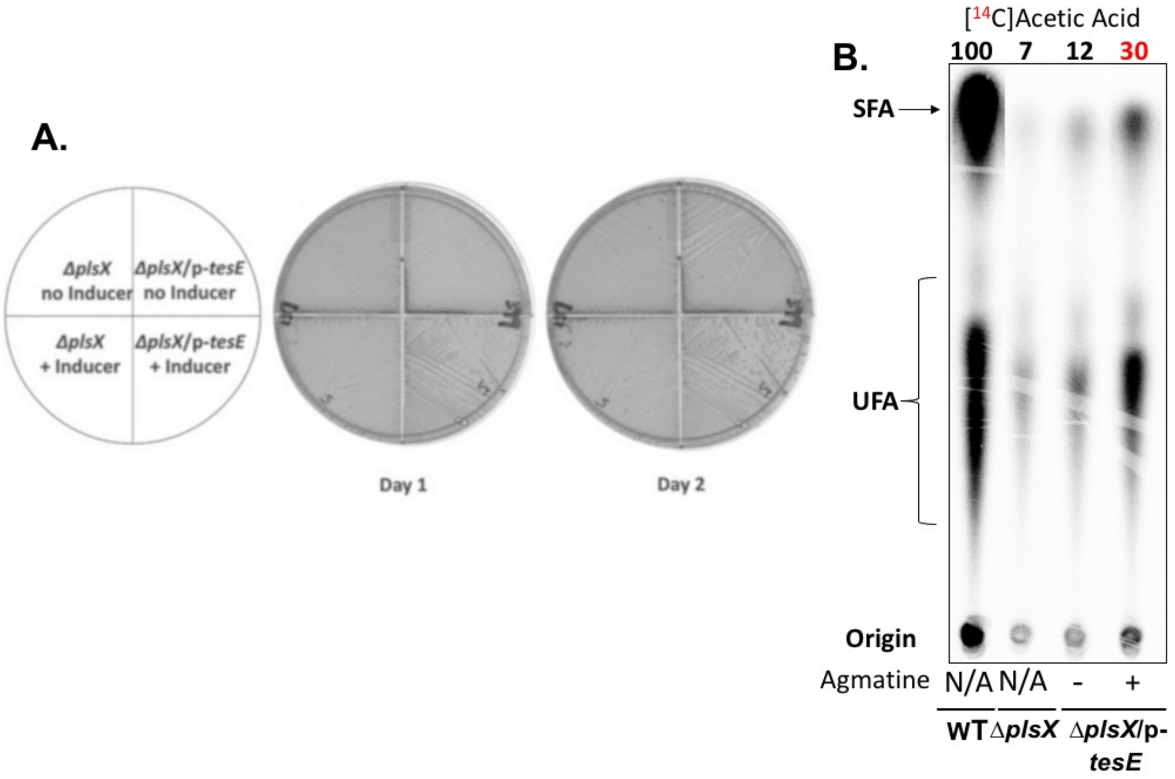

Fig. S3. Induction of TesE overexpression with 5 mM agmatine sulfate allows growth of the Δ*plsX* strain (**A.**) and results in a 2.5-fold increase in acyl chain synthesis (**B.**) Note that the right-hand side of the TLC plate had two additional lanes in which no samples were intentionally loaded that showed very low-level radioactive spots upon phosphorimaging. Since these lanes are an unknown loading artifact, they are irrelevant and are not shown. Similar enhanced phospholipid acyl chain synthesis in the Δ*plsX*/p-*tesE* strain in the presence of the same concentration of agmatine sulphate inducer was also detected in an independent experiment.

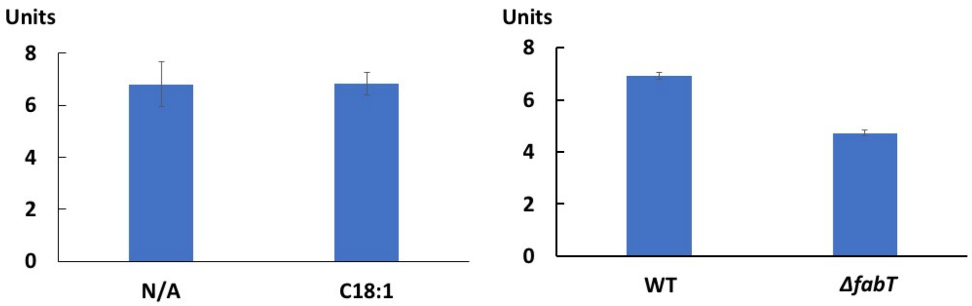

Fig. S4. The expression of *E. faecahe lis tesE* gene is not regulated by exogenous oleate or by FabT and hence is not part of the *E. faecalis* fatty acid synthesis regulon. The left graph shows LacZ activity driven by the *tesE* promoter in the wild-type strain in the absence or presence of oleate whereas in the right graph the LacZ activity driven by the *tesE* promoter of the *E. faecalis* Δ*fabT* strain is less than that of the wild-type strain. The *lacZ* expression from the *tesE* promoter in the wild-type strain in the presence of oleic acid was measured from independent triplicate cultures. The *lacZ* expression from the *tesE* promoter in Δ*fabT* strain was measured from independent triplicate cultures.

References

1. **Zhu, L, Zou, Q, Cao, X, Cronan, JE**. 2019. *Enterococcus faecalis* encodes an atypical auxiliary acyl carrier protein required for efficient regulation of fatty acid synthesis by exogenous fatty acids. mBio **10**:e00577–19.
2. **Zou, Q, Dong, H, Zhu, L, Cronan, JE**. 2022. The *Enterococcus faecalis* FabT transcription factor regulates fatty acid biosynthesis in response to exogeneous fatty acids. Front Microbiol **13**:877582.
3. **Blancato, VS, Magni, C**. 2010. A chimeric vector for efficient chromosomal modification in Enterococcus faecalis and other lactic acid bacteria. Lett Appl Microbiol **50**:542–546.
4. **Bi, H, Zhu, L, Wang, H, Cronan, JE**. 2014. Inefficient translation renders the *Enterococcus faecalis fabK* enoyl-acyl carrier protein reductase phenotypically cryptic. J Bacteriol **196**:170–179.
